# Fine‐Tunable and Injectable 3D Hydrogel for On‐Demand Stem Cell Niche

**DOI:** 10.1002/advs.201900597

**Published:** 2019-07-15

**Authors:** Ki Hyun Hong, Young‐Min Kim, Soo‐Chang Song

**Affiliations:** ^1^ Center for Biomaterials Biomedical research Institute Korea Institute of Science and Technology Seoul 02792 Republic of Korea; ^2^ Division of Bio‐Medical Science and Technology KIST School Korea University of Science and Technology Seoul 02792 Republic of Korea

**Keywords:** β‐cyclodextrin, fine‐tuned, host–guest interaction, stem cell niche, thermosensitive

## Abstract

Stem‐cell‐based tissue engineering requires increased stem cell retention, viability, and control of differentiation. The use of biocompatible scaffolds encapsulating stem cells typically addresses the first two problems. To achieve control of stem cell fate, fine‐tuned biocompatible scaffolds with bioactive molecules are necessary. However, given that the fine‐tuning of stem cell scaffolds is associated with UV irradiation and in situ scaffold gelation, this process is in conflict with injectability. Herein, a fine‐tunable and injectable 3D hydrogel system is developed with the use of thermosensitive poly(organophosphazene) bearing β‐cyclodextrin (β‐CD PPZ) and two types of adamantane‐peptides (Ad‐peptides) that are associated with mesenchymal stem cell (MSC) differentiation and that serve as stoichiometrically controlled pendants for fine‐tuning. Given that complexation of hosts and guests subject to strict stoichiometric control is achieved with simple mixing, these fabricated hydrogels exhibit well‐aligned, fine‐tuning responses, even in living animals. Injection of MSCs in fine‐tuned hydrogels also results in various chondrogenic differentiation levels at three weeks postinjection. This is attributed to the differential controls of Ad‐peptides, if MSC preconditioning is excluded. Eventually, the fine‐tunable and injectable 3D hydrogel could be applied as platform technology by simply switching the types of peptides bearing adamantane and their stoichiometry.

## 1 Introduction

Stem cells constitute an emerging cell source for regenerative tissue engineering owing to their pluripotency, capacity for self‐renewal, and their potential to provide solutions for various human disorders, such as osteoarthritis and cardiac disease.^1^ The potential of stem cell based tissue engineering could be enhanced with increased stem cell retention in designated lesions, enhancement of viability, and enforcement of desired control in their differentiation patterns.^2^ Accordingly, these aforementioned issues are considered as challenges in establishing clinically useful stem cell derived tissue engineering technologies.

Intravenous or intraperitoneal administration of stem cells without use of scaffolds could allow stem cells to be spread to the entire body within a short period.^3^ Therefore, the use of biomaterials for tissue engineering is essential for increased stem cell retention, thereby avoiding repetitive stem cell administration.^4^ To enhance the stem cell retention in a designated lesion site, the use of hydrogels is the best option for stem cell encapsulation owing to their capacity to absorb large amounts of water, their high biocompatibility, and their close resemblance to living tissue.^5^ If injectable hydrogels (e.g., with temperature‐ and pH‐dependent responsiveness) are employed for stem cell derived tissue engineering, considerable advantages will be evoked in terms of the minimal invasiveness and the convenience for recovering irregularly shaped lesions, such as uneven cartilage lesion site in osteoarthritis.^6^ Increased retention of stem cells by using an injectable hydrogel also has resulted in successful tissue engineering outcomes, as reported previously by our group.^7^

The survival rate of administrated stem cells in hydrogel is also a significant issue for superior tissue engineering outcomes.^8^ Stem cell viability could be limited when these suffer immune rejection and unpredictable severe physiological condition even in the injectable hydrogel. Hence, there are several available ways to enhance the stem cell survival rate, such as the use of genetically engineered stem cells,^9^ the employment of bioactive factors,^10^ and the enhancement of biocompatibility of hydrogels.^11^ However, genetic engineering of stem cells is associated with conflicts between the safety and the transduction efficiency when each viral or nonviral vector is used.^9^ Therefore, using bioactive factors and biocompatible scaffolds is a fine choice for the enhancement of stem cell viability, and is free from safety risks.

To obtain an ideal tissue engineering with hydrogels, the fine‐tuning of hydrogel with bioactive factors is required to achieve the desired differentiation patterns of stem cells.^12^ Although there are various types of biocompatible and injectable stem cell scaffolds such as poly(lactic acid) derivate,^13^ hyaluronic acid,^14^ and supramolecular hydrogel,^15^ the controlled differentiation of stem cells is of foremost importance to the attainment of the desired tissue construction. Depending on the use of specific differentiation inducible factors, including chemicals, proteins, and peptide, the fate of stem cells and ultimate phenotype may vary (e.g., stem cells may differentiate into osteocytes, chondrocytes, myocytes, adipocytes, etc.).^16^ Therefore, fine‐tuning the scaffold is essential for the ultimate stem cell differentiation.^17^ Although recently reported fine‐tuned hydrogels were prepared to regulate stem cell differentiation, it is difficult to use them in vivo system owing to the requirement for use of transdermal UV irradiation, which is carcinogenic.^18^ Conversely, from the perspective of fine‐tuning hydrogels with bioactive molecules, the modification of hydrogel for the stem cell differentiation leads to unpredictable molecular substitutions^19^ or physical mixing of bioactive factors.^20^ In the construction of fine‐tunable and injectable hydrogels, the polymeric pendant modification with at least two types of bioactive molecules is also difficult without (meth)acrylate‐/UV‐mediated conjugation, which permits only in vivo transplantation.^21^ This means that the preparation of a fine‐tuned and injectable hydrogel as a potential stem cell derived tissue engineering approach is associated with a conflict. Eventually, to fulfill the fine‐tuning of bioactive molecules and maintain the innate injectability of the hydrogel, host–guest interactions, noncovalent systems, could become wonderful alternatives compared to the use of a chemical conjugation system.

To address these important issues, such as the retention of stem cells, stem cell viability, and the control of stem cell differentiation, we propose an integrated concept that accounts for the fine‐tuning and injectability issues of 3D stem cell scaffolds. To design a fine‐tunable and injectable 3D hydrogel to serve as stem cell scaffolds, we employed a hybrid system based on host–guest interactions and thermosensitive hydrogels. Given that/st interactions exhibit the strong self‐assembly characteristics between host and guest molecules, the fine‐tuning process is achieved by controlling the stoichiometric ratio of guest molecules. Among the various host molecules, such as cyclodextrins, cyclophanes, and curcubiturils, β‐cyclodextrin (β‐CD) is selected as the host owing to its high water solubility postmodification, low toxicity, and low immunogenicity.^22^ Since a guest molecule of adamantane (Ad) exhibits a high binding affinity with β‐CD, it is also chosen as the guest molecule,^22^ and is elongated with two types of stem cell differentiation induction peptides (Ad‐peptide). Despite the existence of many studies on the topic of host–guest interaction using β‐CD and Ad, their roles were limited in the gelation process based on host–guest interaction and the pendantly attached guest molecule carrier.^23^ Beyond these issues, we approach the host–guest interaction as a means of fine‐tuning with maintaining injectability of hydrogel. Consequently, fine‐tuned hydrogels fabricated by the host–guest interaction based on simple mixing did not require further chemical synthesis processes.

**Figure 1:**
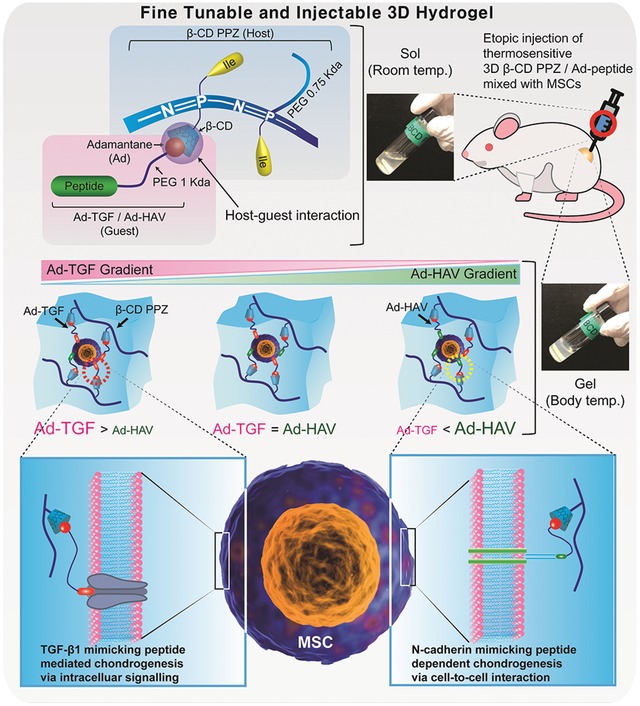
Schematic representation of the injection of mesenchymal stem cells (MSCs) encapsulated with 3D β‐cyclodextrin poly(organophosphazene) (β‐CD PPZ), adamantine‐TGF, and HAV (Ad‐TGF and Ad‐HAV). Aforementioned several molecules and cells were simply fabricated to form 3D thermosensitive hydrogels. After injecting them once, the solution containing the indicated mixture was transformed into a solidified gel. To control the fate of MSCs, Ad, guest molecule for β‐CD, which was elongated with TGF‐β1 peptides (Ad‐TGF) and N‐cadherin peptides (Ad‐HAV) was introduced, and it exhibited stoichiometric flexibility in this 3D hydrogel.

For several decades, various kinds of thermosensitive hydrogels (e.g., poly(*N*‐isopropylacrylamide), Pluronics, or Poloxamer) had been developed tremendously in the field of tissue engineering. However, the limitations of aforementioned hydrogels had also been reported such as nonbiodegradibility, toxicity, and lack of functional group.^24^ Hence, in this study, the injectable hydrogel was originated from poly(organophosphazene) (PPZ), which benefits for biocompatibility, biodegradability, and thermosensitivity. Once we succeeded in synthesizing both the thermosensitive PPZ bearing β‐CD (β‐CD PPZ, host) and two types of mesenchymal stem cell (MSC) chondrogenesis inducing Ad‐peptides (guests, bioactive peptides derived from TGF‐β1 and N‐cadherin), the fine‐tuning process was conducted based on the stoichiometric control of Ad‐peptides in β‐CD PPZ (**Figure** **1**). Fine‐tuned hydrogels were prepared via simple mixing, and incorporated with various stoichiometric ratios of Ad‐peptides without any additional synthetic processes. Furthermore, various degrees of MSC differentiation were resulted from the control of the Ad‐peptide ratio. This system could be utilized as a platform technology by switching the types of guest molecules or their stoichiometry to achieve the fine‐tunable and injectable 3D construct.

## 2 Results and Discussion

### 2.1 Manufacturing β‐CD PPZ and Ad‐Peptides

To synthesize β‐CD PPZ, the host molecule, PPZ that contained —COOH (acid PPZ) at the terminal end was prepared as described in our previous report.^25^ Briefly, the thermally ring‐opened poly(dichlorophosphazene) was reacted with isoleucine ethyl ester (IleOEt), α‐amino‐ω‐methoxy‐poly(ethyleneglycol) (AMPEG), and 2‐aminoethanol in order under the completely moistureless condition. The intermediate PPZ product that contained the hydroxyl group (—OH) from 2‐aminoethanol was amphiphilic, owing to the retention of both hydrophilic AMPEG and hydrophobic IleOEt. This amphiphilic property of PPZ had aided the thermosensitive sol–gel transition behavior.^26^ The PPZ exposing —OH groups were esterified to yield the —COOH groups, by allowing them to react with glutaric anhydride, and these —COOH groups were then conjugated with the primary amine (—NH_2_) containing β‐CD, to allow the formation of β‐CD PPZ via 1‐ethyl‐3‐(3‐dimethylaminopropyl) carbodiimide (EDC) chemistry (Scheme S1, Supporting Information). The structure of β‐CD PPZ and its accurate conjugation, which have been confirmed with ^1^H‐NMR and Fourier transform infrared spectroscopy (FT‐IR), were shown in Figure S1 in the Supporting Information. Eventually, each PPZ containing —COOH and β‐CD showed a thermosensitive sol–gel transition (Figure S2, Supporting Information). The diminution of *T*_0_, the temperature that exhibited the initially increased viscosity, has been observed in the viscosity result of β‐CD PPZ, as compared to that of acid PPZ. This phenomenon had been caused by the decrease in the hydrophilic —COOH substitution to β‐CD. Furthermore, the chemical evidence for β‐CD conjugation was obtained by ^1^H‐NMR measurement of the β‐CD conjugation specific anomeric proton generation; the FT‐IR peak change was observed because of C=O stretching in β‐CD PPZ, as compared to that in acid PPZ.

Bioactive peptides derived from proteins could be a great alternative, after addressing the drawbacks resulting from the use of proteins, which exhibits a structural instability. We had chosen two different tracks for inducing MSC chondrogenesis, i.e., stimulation of receptor mediated signaling (TGF‐β1) and cell‐to‐cell interactions (N‐cadherin). TGF‐β1 exists at the site of embryonic bone and cartilage development, and has a critical role in the intracellular signaling cascade that facilitates cartilage‐specific gene expression.^27^ Out of all the peptide sequences in TGF‐β1, the binding site exhibiting the maximum reactivity to the TGF‐β1 receptor was selected for this study (CESPLKRQ).^28^ The next protein source is N‐cadherin, which has a significant role in both cell‐to‐cell interaction and chondrogenesis. In a recent decade, the His‐Ala‐Val (HAV) motif derived from N‐cadherin has been mentioned in many studies, owing to its performance during chondrogenesis.^18^, ^29^ Though the HAV sequence alone is enough to induce MSC chondrogenesis, a previous study revealed that an elongated HAV sequence such as CLRAHAVDIN was more effective than the short HAV motif.^30^ These two kinds of selected proteins are commonly associated with both mitogen‐activated protein kinase (MAPK) signaling and the regulation of its subunits such as p38, extracellular signal‐regulated kinase‐1, and c‐Jun N‐terminal kinase, with regard to MSC chondrogenesis.^31^ We prepared a couple of Ad‐peptides, by mimicking them from TGF‐β1 and N‐cadherin (Ad‐TGF and Ad‐HAV) (Scheme S2, Supporting Information). Adamantane acetic acids were elongated with 1.0 kDa of diamine poly(ethylene glycol) (PEG), to produce monoamine adamantane PEG (Ad‐PEG‐NH_2_). The byproducts of diadamantane PEG and PEG were eliminated using column chromatography. Such an Ad‐PEG‐NH_2_ product was modified with methacrylate chloride, to yield methacrylate groups (Figure S3, Supporting Information). Ad‐TGF and Ad‐HAV were prepared through the Michel click chemistry between methacrylate containing adamantane and Cys (—SH) containing peptide sequences. These final products of Ad‐TGF and Ad‐HAV were characterized using ^1^H‐NMR spectra. Their peptide specific guanidine and amide bond peaks of Ad‐TGF and Ad‐HAV were observed in ^1^H‐NMR spectra (Figures S4 and S5, Supporting Information).

**Figure 2:**
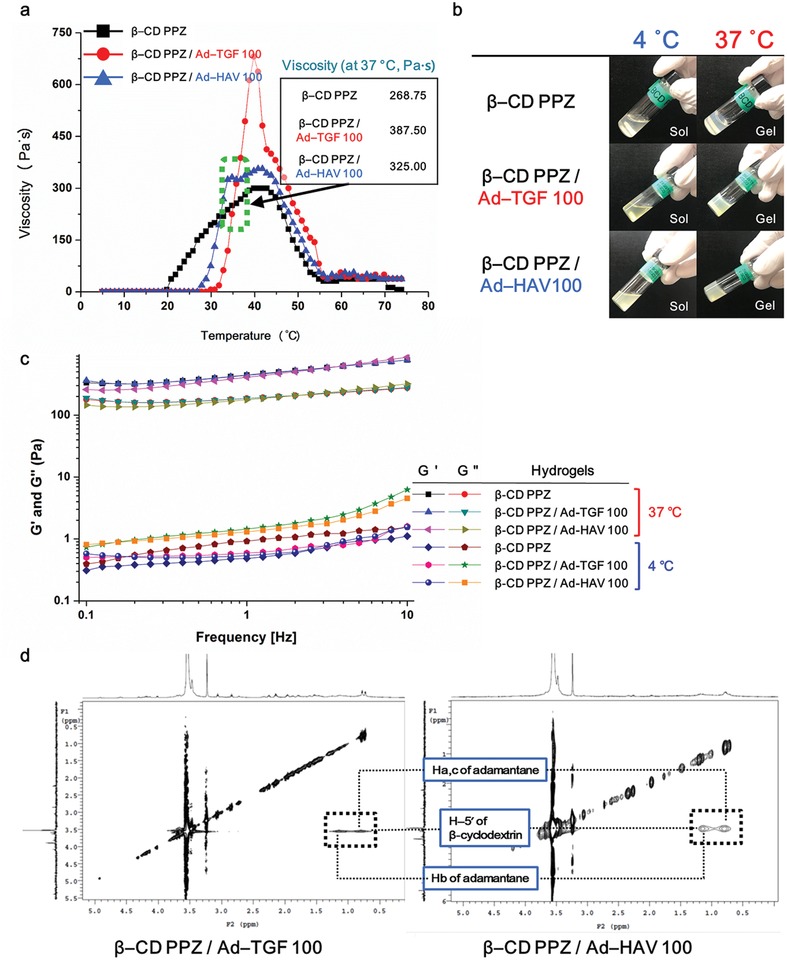
Fabrication of thermosensitive β‐CD PPZ/Ad‐TGF or HAV hydrogels, based on their assembly using host–guest interactions. β‐CD PPZ in all groups was dissolved with PBS buffer for the measurements of viscosity and rheology at the concentration of 10 wt%. a) Thermosensitive gelation details for β‐CD PPZ, β‐CD PPZ/Ad‐TGF 100, and β‐CD PPZ/Ad‐HAV 100. Viscosities at the body temperature (37 °C) were also elucidated for each group. b) Sol–gel transition visualization for β‐CD PPZ (top), β‐CD PPZ/Ad‐TGF 100 (middle), and β‐CD PPZ/Ad‐HAV 100 (bottom). c) The storage modulus (*G*′) and loss modulus (*G*″) of β‐CD PPZ, β‐CD PPZ/Ad‐TGF 100, and β‐CD PPZ/Ad‐HAV 100 at 4 and 37 °C for displaying sol–gel transition. d) 2D‐NOESY results that provide evidence of the occurrence of host–guest interactions between β‐CD PPZ and Ad‐TGF (left), and β‐CD PPZ and Ad‐HAV (right).

Following the synthesis of host and guest molecules, the temperature‐dependent gelation profiles and the visualization of sol–gel transition with β‐CD PPZ molecules, which contained Ad‐TGF 100 and Ad‐HAV 100, were measured. The numbering of the following Ad‐peptides is associated with the Ad‐peptide contents percentage in relation to that of the entire molecules of β‐CD in β‐CD PPZ. For instance, Ad‐TGF 50 represents the presence of 50% saturated Ad‐TGF, as compared to that of fully saturated Ad‐TGF, in the inclusion complex with β‐CD. Furthermore, we had observed the viscosity properties of our fabricated hydrogels. Even if Ad‐TGF 100 and Ad‐HAV 100 were incorporated in β‐CD PPZ, both hydrogels had shown gelation properties and a sufficient level of viscosity, which impacted the body temperature (**Figure** **2**a,b). *T*_0_ values of β‐CD PPZ/Ad‐TGF 100 and β‐CD PPZ/Ad‐HAV 100 after fabrication, achieved via host–guest interactions, were slightly higher than viscosity of β‐CD PPZ only, owing to the possession of hydrophilic PEGs in Ad‐TGF and HAV. In particular, β‐CD PPZ, β‐CD PPZ/Ad‐TGF 100, and β‐CD PPZ/Ad‐HAV 100 hydrogels showed a certain viscosity at body temperatures of 268.75, 387.50, and 325.00 pa s, respectively (Figure 2a). Furthermore, the loss modulus (*G*″) was higher than the storage modulus (*G*′) at a cool temperature (4 °C) whereas *G*′ value was greater than *G*″ at a body temperature in the rheological results, irrespective of any hydrogels (Figure 2b). It means that the sol state at cool temperature was changed to the gel state in all of the hydrogels by increasing a temperature up to 37 °C. Based on these proofs for the thermosensitive property from the viscosity and rheology results, all of the fabricated hydrogels were suitable for the administration into live animals.

### 2.2 Establishment of Fine‐Tuned and Thermosensitive Hydrogels Consisting of β‐CD PPZ and Ad‐Peptides

**Figure 3:**
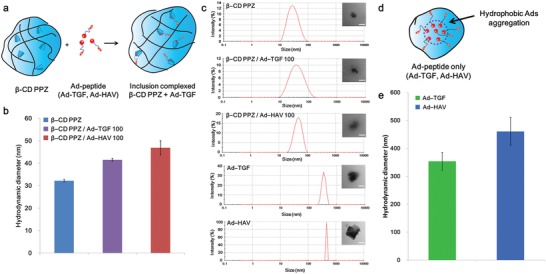
Host–guest interactions between β‐CD PPZ (host) and Ad‐peptides (guest). a) The illustration showing host–guest interactions between β‐CD PPZ and Ad‐peptides. b) Hydrodynamic diameter results for β‐CD PPZ, β‐CD PPZ/Ad‐TGF, and Ad‐HAV (*n* = 3). c) Size distribution results and TEM images of β‐CD PPZ, β‐CD PPZ/Ad‐TGF, Ad‐HAV, and Ad‐peptides alone (Ad‐TGF and Ad‐HAV). Scale bar is 50 nm for β‐CD PPZ, β‐CD PPZ/Ad‐TGF, and Ad‐HAV, and 500 nm for Ad‐peptides alone. d) The illustration showing the aggregation of guest molecule alone in the aqueous state. e) Hydrodynamic diameter results of Ad‐peptides alone (*n* = 3).

To verify the host–guest interactions between β‐CD PPZ and Ad‐peptides, 2D nuclear Overhauser effect spectroscopy (NOESY) spectra were measured in the aqueous state with D_2_O, in the same conditions as those of fabricated hydrogels. 2D‐NMR is an effective method for observing the intermolecular interactions and (or) the conformation of the inclusion complex.^32^ Cross peaks in 2D‐NOESY could be obtained from nuclei resonance connections that were spatially closer than those of the coupled bond.^33^ Cross peaks in 2D‐NOESY spectra that showed the involvement of molecules in host–guest interactions; Ha, c, Hb (δ 0.6–1.3 ppm) of adamantane and H‐5′ (δ 3.4–3.5 ppm) of the β‐CD inner cavity, are fairly elucidated in Figure 2c. Furthermore, to identify whether these guest molecules of Ad‐peptides had stoichiometrically and accurately been inserted into host molecules, dynamic light scattering (DLS) measurement were performed after setting the host:guest ratio = at 1:1, 1:1.2, and 0:1. Above all, the increased hydrodynamic diameters for β‐CD PPZ complexed with Ad‐peptides (host:guest = 1:1) are demonstrated in **Figure** **3**b. The DLS results of using host:guest ratio of 1:1 and any peak involved in the measurement of the guest molecules (Ad‐TGF and Ad‐HAV) alone were not shown (Figure 3c). This means that there were no breakaway guest molecules from β‐CD PPZ. Since the particle sizes of Ad‐TGF and Ad‐HAV alone (host:guest = 0:1) were 353.63 ± 31.71 and 460.78 ± 49.53 nm, respectively (Figure 3d,e), which were much higher size than β‐CD PPZ alone, this phenomenon was resulted from the aggregation of hydrophobic adamantane molecules in the aqueous state. To sum up, all Ad‐peptides had been incorporated into the inclusion complex with β‐CD after complexing of β‐CD PPZ and Ad‐peptides (host:guest = 1:1) in an aqueous environment. On the other hand, when excessive amounts of Ad‐peptides were mixed with β‐CD PPZ (host:guest = 1:1.2), the surplus Ad‐peptides formed other aggregates, whose occurrence was supported by the measurement of analogous peaks, while obtaining DLS results with Ad‐TGF and Ad‐HAV alone (Figure S6, Supporting Information). If there is no host–guest interaction between β‐CD PPZ and Ad‐peptides, other peaks for Ad‐peptide aggregates of DLS could be produced. Consequentially, stoichiometrically controllable Ad‐peptides based on host–guest interaction were proved using both 2D‐NOESY and DLS measurement.

**Figure 4:**
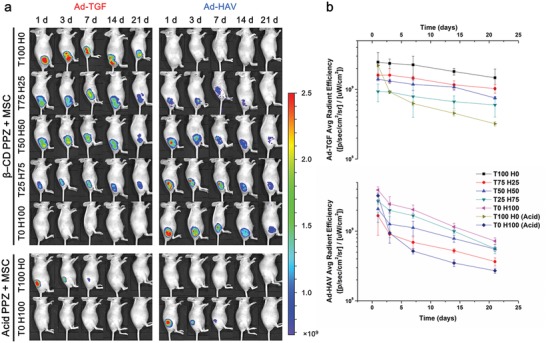
In vivo long‐term maintenance of host–guest interaction dependent on the gradient Ad‐peptides ratio was observed using IVIS. a) Images showing host–guest interacted Ad‐TGFs and Ad‐HAVs in mice; β‐CD PPZ and MSCs tagged with FITC and Rho, respectively. Within a period of 21 days, the gradient fluorescence levels of Ad‐TGFs and Ad‐HAVs were detected using IVIS. b) The average radiant efficiency values in mice Ad‐TGF (top) and Ad‐HAV (bottom) during 21 days (*n* = 3).

We measured the long‐term maintenance of host–guest interaction with initially different amounts of incorporated Ad‐TGF and Ad‐HAV in β‐CD PPZ using an in vivo imaging system (IVIS) in living animals. In the molecular tails of Ad‐TGF and Ad‐HAV, rhodamine (Rho) and fluorescein isothiocyanate (FITC), respectively, were linked by using the conjugation process. These fluorescence expressing guest molecules were then injected after the fabrication of inclusion complexes with β‐CD PPZ. Since β‐CDs in PPZ were lost to the outside region of the 3D hydrogel, owing to PPZ capacities of biodegradability and dissolution of PPZ, Ad‐peptide signals containing fluorescence became lesser over time. Furthermore, the stoichiometric controlled patterns of Ad‐TGF and Ad‐HAV were observed in results during 21 days, even with the use of two different guest molecules (**Figure** **4**a). For instance, the fluorescence intensities of Ad‐TGF were found to be in the increasing order for T100 H0, T75 H25, T50 H50, and T25 H75. This result was derived from stoichiometrically different guest molecules and was dependent on the host–guest interactions. Additionally, the region of interest (ROI) value for the expressed fluorescence was calculated as shown in Figure 4b. As Ad‐peptides were mixed with acid PPZ (lack of β‐CD in PPZ), the rapid escape of Ad‐peptides from 3D hydrogels occurred within day 7. Although the ROI values decreased in processes over time in all the groups (T100 H0, T75 H25, T50 H50, T25 H75, and T0 H100), owing to the biodegradability and dissolution of β‐CD PPZ, the controlled level of Ad‐peptide fluorescence expression in β‐CD PPZ had been produced by initially adding different Ad‐peptides in all groups and at all time points, due to host–guest interactions. This result showed that host–guest interactions in our 3D β‐CD PPZ hydrogel were maintained for a considerably long period even in living animals.

### 2.3 Fine‐Tuned and Injectable Hydrogels for In Vivo Ectopic Chondrogenesis

**Figure 5:**
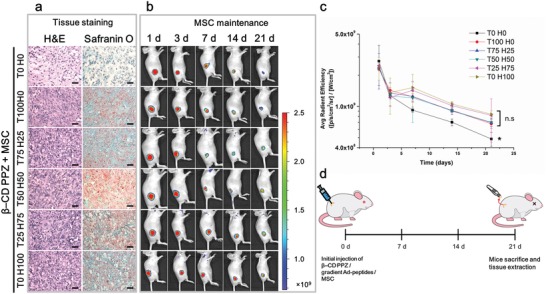
Biocompatibility and basic chondrogenesis capacity using the inclusion complex of β‐CD PPZ/various gradient Ad‐peptides/MSCs. a) The result of H&E staining (left) for biocompatibility verification and safranin‐O staining (right) for the demonstration of chondrogenic differentiation with MSCs (Scale bar = 100 µm). b) The maintenance of MSCs in thermosensitive β‐CD PPZ with various gradient Ad‐peptides measured using IVIS. c) The average radiant efficiency values of GFP tagged MSC in mice during 21 days (*n* = 3). The * on the bar graph indicates the significance (*: *p* < 0.05) of less fluorescence in T0 H0 compared with other groups. d) The basic illustration of the in vivo experimental schedule.

To confirm the biocompatibility of the host molecule of β‐CD PPZ, the in vitro cytotoxicity tests were performed with the 2D plated cells and 3D hydrogel encapsulated cells. The viabilities of 2D plated MSCs treated with 1 wt% hydrogel and MSCs laden 10 wt% 3D hydrogel had been evaluated as 82.3% and 91.2%, respectively (Figure S7, Supporting Information). We assumed that the enhanced interaction between MSCs and biocompatible hydrogel had induced the superior MSC viability in the 3D hydrogels. Furthermore, the previous studies also had demonstrated that the viability of stem cells encapsulated in 3D hydrogel was greater than 2D plated stem cells.^34^ It suggests that this polymer showed a promising level of biocompatibility for the next study, which would be conducted in animal test. Basically, the in vivo biocompatibility of postfabrication with β‐CD PPZ, and use of ratio‐controlled Ad‐peptides and MSCs was confirmed using hematoxylin and eosin (H&E) staining. According to the histological results obtained after H&E staining, any foreign body responses such as the generation of foreign‐body giant cells and toxicity were not observed in all groups of MSCs encapsulated with β‐CD PPZ/ratio‐controlled Ad‐peptides (**Figure** **5**a). The neo‐chondrogenesis occurring in ectopically injected MSCs encapsulated with β‐CD PPZ/ratio‐controlled Ad‐peptide was then observed using safranin‐O staining. Ectopically generated cartilages and cytoplasm were stained in red and green color, by safranin‐O and fast green, respectively. As shown in Figure 5a, the remarkable levels of cartilage stained colors as red were observed in Ad‐peptides contained groups. However, T0 H0 group had shown no staining of the cartilage, owing to the absence of peptide stimulation for chondrogenesis. Here, we showed that the qualitative analysis of chondrogenesis was performed using safranin‐O staining under conditions of stoichiometric control of Ad‐peptides. All Ad‐peptides incorporating groups had exhibited the MSC chondrogenic differentiation. Our results from safranin‐O staining, which showed occurrence of ectopically induced chondrogenesis, were similar to a previous study by another group and even their treatment of the chondrogenic induction media.^13^

Moreover, in vivo MSC maintenance was measured using IVIS for the reason that the local presence of MSCs over long‐term remained MSCs enhanced their therapeutic efficiency.^35^ Hence, MSCs capable of expressing green fluorescent proteins (GFPs) were enveloped within the inclusion complexed β‐CD PPZ/Ad‐peptides, to measure the long‐term MSC maintenance in living animals. Locally injected MSCs were maintained at their injection sites for a period of 21 days. However, the significantly low maintenance of MSC fluorescence was monitored in T0 H0 group with an absence of both receptor‐mediated MSC attachment and factors promoting cell‐to‐cell interactions (Figure 5b). Previous reports showed that cell‐to‐cell interactions and TGF‐β1 stimulation can enhance the survival rate of stem cells as well as chondrogenic differentiation.^36^ The continuous decline of GFP tagged MSC in all groups resulted from biodegradability of β‐CD PPZ. Nevertheless, the considerably reduced level of MSC maintenance was shown in T0 H0 group, owing to an absence of both cell‐to‐cell interaction and TGF‐β1 stimulation. Evaluation of ROI value for the remained MSCs also substantiated the low level MSC maintenance in T0 H0, as compared to that in other Ad‐peptides containing groups (Figure 5c). The presence of Ad‐peptides with MSCs had assisted local MSC maintenance at injected sites. In summary, these guest molecules, which were stoichiometrically incorporated in β‐CD PPZ hydrogels, were biocompatible at both in vitro and in vivo levels. Moreover, MSC chondrogenesis induction using β‐CD PPZ/Ad‐peptide complex was also observed in all groups except T0 H0 group. In the next section, we had thoroughly evaluated the chondrogenic differentiation levels in an environment with stoichiometrically controlled Ad‐peptides.

**Figure 6:**
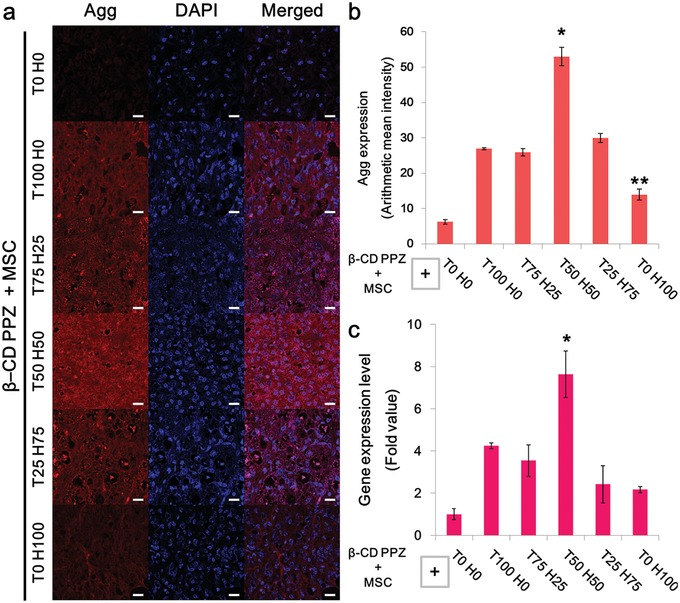
MSC chondrogenic induction screening with the various combinations of Ad‐peptides with β‐CD/MSCs. Mice were sacrificed at day 21 postinjection. a) Immunohistochemistry analysis for Agg, the representative protein used for chondrogenesis detection (Scale bar = 20 µm). b) Analysis of fluorescence intensities of Agg (*n* = 3). c) Agg gene expression levels in mice tissues (*n* = 3). The * on the bar graph indicates the significance (*, **: *p* < 0.05) of Agg expression in T50 H50 compared with other groups (*) and less protein expression compared with T100 H0, T75 H25, T50 H50, and T25 H75 (**).

**Figure 7:**
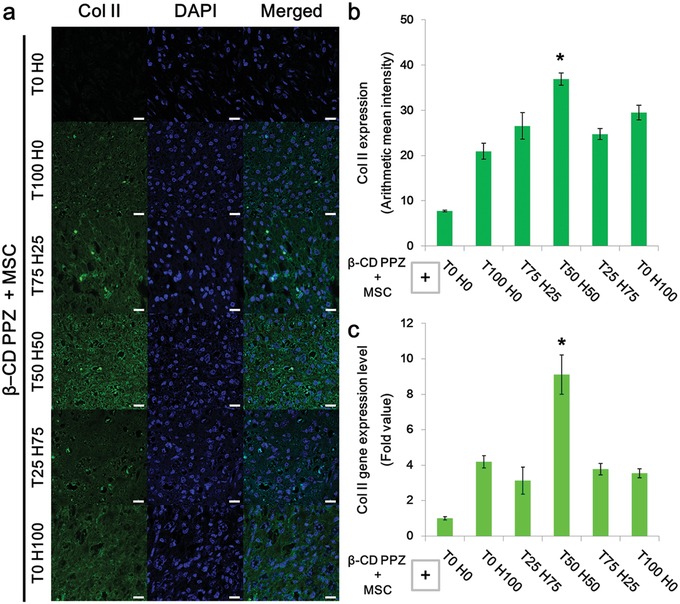
MSC chondrogenic induction screening with the various combinations of Ad‐peptides with β‐CD/MSCs. Mice were sacrificed at day 21 postinjection. a) Immunohistochemistry analysis for Col II, the representative protein used for chondrogenesis detection (Scale bar = 20 µm). b) Analysis of fluorescence intensities of Col II (*n* = 3). c) Col II gene expression levels in mice tissues (*n* = 3). The * on the bar graph indicates the significance (*: *p* < 0.05) of Col II expression in T50 H50 compared with other groups.

Evaluation of exact levels of MSC chondrogenesis using the flexible, varied different guest molecule compositions had been performed with typically used chondrogenesis markers of aggrecan (Agg) and type‐II collagen (Col II). The consequences of chondrogenesis were evaluated by measuring the gene expression levels and by immunohistochemistry. Ectopically neo‐formed tissues resulted from the local and subcutaneous injection of MSCs encapsulated with β‐CD PPZ and Ad‐peptides were extracted in all experiment groups. Agg, the cartilage‐specific proteoglycan core protein, was fairly synthesized and expressed in all the Ad‐TGF and/or Ad‐HAV incorporating groups (**Figure** **6**a). In particular, the highest Agg specific fluorescence and gene expression level were observed in T50 H50 compared to T100 H0, T75 H25, T25 H75, and T0 H100 (Figure 6b,c). The level of gene and protein expression in T50 H50 is almost twice compared to other Ad‐peptide involving groups such as T100 H0, T75 H25, and T25 H75. In the absence of Ad‐peptides, there was no significant Agg expression during the measurement of both gene and protein fluorescence. Col II, the major protein in cartilage, was also selected as the specific marker for evaluating chondrogenesis. Col II was well developed under the incorporation of Ad‐peptides in β‐CD PPZ compared to T0 H0 group (**Figure** **7**a). The expressed gene and fluorescence levels of Col II were also similar to the Agg expression pattern as shown in Figure 7b,c. Notably, the highest expressions of Col II fluorescence and gene were shown in T50 H50 group. Because the contrary protein expression levels for Agg and Col II were observed in T0 H100 group, we assumed that TGF‐β1 alone could upregulate Col II expression. After all, the MSCs encapsulated with β‐CD PPZ and fine‐tuned Ad‐peptides had resulted in distinct levels of chondrogenesis. Among all of the groups, the highest levels of typical chondrogenesis markers were identified in T50 H50 group.

Fundamentally, TGF‐β1, which orchestrates the elaborate control of MAPK factors is significantly associated with the initiation of MSC chondrogenesis.^37^ N‐cadherin had also notably induced the chondrogenesis of MSC via cell‐to‐cell interactions.[qv: 29a,38] While different routes by using both Ad‐peptides were employed for inducing MSC chondrogenesis in this study, MAPK activation for the chondrogenic differentiation had been harmonized by both TGF‐β1 and N‐cadherin.^31^ As aforementioned, MSC differentiations with TGF‐β1 and N‐cadherin are concentrated on the intracellular signaling or cell‐to‐cell interaction mediated chondrogenesis, respectively. Hence, the moderate chondrogenesis was resulted from the perfectly leant Ad‐peptide treatment groups such as T100 H0 and T0 H100 owing to the deficiency of opposite stimulation. Though T75 H25 and T25 H75 groups were treated with both kinds of Ad‐peptides, the modest levels of chondrogenesis had also been produced due to the one‐side strong Ad‐peptides MAPK stimulation. Outstanding levels of chondrogenesis were generated by fairly balanced Ad‐peptides group of T50 H50, which simultaneously stimulated MAPK factors. We assumed that well‐balanced MAPK stimulation in MSCs was stronger than the slanted Ad‐peptide stimulation for MSC chondrogenesis such as groups of T100 H0, T75 H25, T25 H75, and T0 H100. Previous studies had shown that the inductions of chondrogenesis were under the treatment of ≈10–20 000 times higher concentrations of TGF‐β1 or N‐cadherin than our T50 and (or) H50 Ad‐peptide employment.^31^, ^39^ We assumed that stimulation with both Ad‐peptides at concentrations of 1.23 × 10^−13^ mole per cells (in T50 H50) had been sufficient for inducing MSC chondrogenesis even at the lower Ad‐peptide concentrations in our study. From these results, it can be observed that none of the factors was dominant; instead they were cooperative for the development in chondrogenesis. Eventually, these balanced two Ad‐peptides group such as T50 H50 had elicited an optimal level of chondrogenesis in this system.

### 2.4 Fine‐Tuned and Injectable Hydrogels for Avoiding Osteogenesis

**Figure 8:**
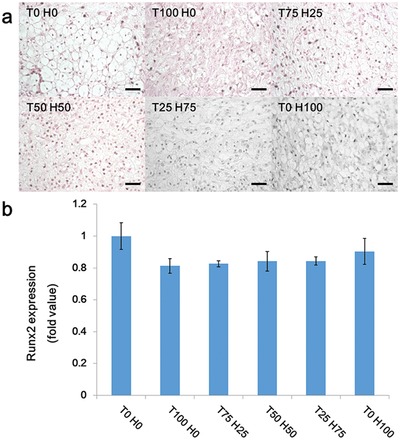
Analysis of the evidence of nonosteogenesis of each Ad‐peptide combinations injection with β‐CD PPZ and MSCs after 21 days. a) Von Kossa staining with various Ad‐peptide compositions/β‐CD PPZ/MSCs (Scale bar = 100 µm). b) Runx2 gene expression levels, the typical gene marker for osteogenesis, was observed for the analysis of osteogenesis (*n* = 3).

Osteogenesis, also known as ossification, is a single continuous development process, for which cartilage formation acts as a precursor.^40^ Thus, even if hypertrophic chondrocytes are differentiated into osteocytes in the presence of specific stimulators such as runt‐related transcription factor 2 (Runx2) and osterix, the use of Ad‐peptides should prevent the further process of chondrocyte to osteogenic termination.^41^ It is important that the inhibition of osteogenesis occurs only because of the generation of incorrect terminal MSC differentiation products during the chondrogenesis process. Generally, Runx2 is considered as the typical key transcription factor for osteogenesis. Hence, Runx2 was a candidate gene assay marker for detecting the further progression of osteogenesis. Furthermore, we had tried to observe the calcium production, a significant marker for a final osteogenesis product, in the collected tissues via von Kossa staining. There was no calcium dot observed to be involved in any experimental group (**Figure** **8**a). Furthermore, considerably excessive amounts of Runx2 genes were not detected in any groups, as compared to T0 H0 group (Figure 8b). By all accounts, the final product of osteoblast development, such as calcium and Runx2, were clearly not observed. Eventually, the occurrence of osteogenesis was completely inhibited even using TGF‐β1 derived peptide in this study.

## 3 Conclusion

We showed that injectable 3D hydrogel fine‐tuned with diverse peptides and its proportions had induced MSCs chondrogenesis. 3D MSC niche was constructed using one host molecule (β‐CD PPZ) and two kinds of guest molecules (Ad‐TGF and Ad‐HAV). Guest molecules in 3D hydrogel were under the stoichiometrically strict control based on host–guest interaction. Although these Ad‐peptides were formed in an inclusion complex with β‐CD PPZ, their gelation properties were still maintained at the body temperature. These fabricated hydrogels, which incorporated various ratios of Ad‐TGF/Ad‐HAV and MSCs, were subcutaneously injected into pockets of living animals to induce chondrogenesis. As two Ad‐peptides had been used to induce MSC chondrogenesis via different routes, a remarkable level of chondrogenesis was observed in T50 H50 group with stoichiometrically balanced Ad‐peptides, owing to balanced stimulation of MAPK. Furthermore, we showed that β‐CD PPZ/Ad‐peptide mediated chondrogenesis did not induce osteogenesis, as posthypertrophic stage of MSCs. We had performed the MSC differentiation process, independently of the method for MSC preconditioning, such as the employment of induction media. Nonetheless, one advance in this study is the facile fabrication of hydrogel with controllable guest molecule without further synthesis steps. Diverse peptides bearing adamantane, which are available for host–guest interactions, could be utilized in our system. Eventually, this technology could be used as a platform system by switching kinds of peptides bearing adamantane or their ratios to manufacture ideal 3D biomedical constructs.

## 4 Experimental Section

*Materials*: Hexachlorocyclotriphosphazene (Aldrich) was purified by sublimation at 55 °C under vacuum (about 0.1 mmHg). Poly(dichlorophosphazene) was prepared as described previously.^42^ It was prepared from hexachlorocyclotriphosphazene using aluminum chloride (AlCl_3_) as a catalyst at 250 °C during 5 hr. l‐Isoleucine ethyl ester hydrochloride (IleOEt⋅HCl) was prepared from l‐isoleucine (Aldrich) according to the literature. α‐Amino‐ω‐methoxy‐poly(ethylene glycol)s with molecular weights of 750 Da were prepared according to the literature.^43^ Tetrahydrofuran (THF) and triethylamine (TEA) (Junsei Chemical Co., Ltd.) were purified under the dry nitrogen atmosphere by refluxing at the boiling point over sodium metal/benzophenone (Acros) and barium oxide (Acros). β‐Cyclodextrin purchased from Aldrich was used without further purification. Mono‐6‐OTs‐βCD and mono‐6‐diethylamino‐βCD (NH_2_‐βCD) were synthesized according to the method reported in the literature. Acetonitirile (ACN), ethanol amine (AEtOH), 4‐(dimethylamino) pyridine (DMAP), isobutyl chloroformate (IBCF), and 1‐ethyl‐3‐(3‐dimethylaminopropyl) carbodiimide were obtained from Aldrich. Dichloromethane (DCM) was purchased from Daejung Chemical Company (Korea) with an extra pure quality and no further purification. N‐cadherin mimic peptide (CLRAHAVDIN) and TGF‐β1 mimic peptide (CESPLKRQ) were purchased from LifeTein, LLC (US).

*Synthesis of β‐Cyclodextrin Conjugated Poly(oranophosphazene) (β‐CD PPZ)*: All reactions were processed under a dry nitrogen atmosphere using standard Schlenk‐line techniques. β‐CD PPZ was the same as that used in the previous study and the detailed synthesis protocol can be found in the paper. Briefly, IleOEt, AEtOH, and AMPEG750 in dried THF were added slowly to poly(dichlorophosphazene) in dried THF/TEA. After purification, glutaric anhydride and 4‐(dimethylamino) pyridine (DMAP) in dried THF were also added to the poly(dichlorophosphazene) solution to transform the hydroxyl group of AEtOH to carboxyl group. Finally, mono‐6‐diethylamino‐β‐CD was conjugated to the activated carboxyl group of PPZ using 1‐ethyl‐3‐(3‐dimethylaminopropyl) carbodiimide. Purification of synthesized β‐CD PPZ was performed using dialysis and lyophilization.

*Synthesis of Ad‐PEG‐CLRAHAVDIN (Ad‐HAV) and Ad‐PEG‐CESPLKRQ (Ad‐TGF)*: Ad‐PEG‐MeAc (*M*_W_: 1302.6 Da, 200 mg, 0.15 mmol) was dissolved in pH 10.0 adjusted aqueous solutions. Tris (2‐carboxyethyl) phosphine) (TCEP, 44.0 mg, 0.15 mmol) and TGF‐β1 mimic peptide (CESPLKRQ, 960.12 Da, 442.2 mg, 0.46 mmol) were added to Ad‐PEG‐MeAc solution at once. In case of Ad‐HAV, N‐cadherin mimic peptide (CLRA**HAV**DIN, 1111.29 Da, 767.8 mg) were added to Ad‐PEG‐MeAc solution. Reaction solution was purged by N_2_ atmosphere for 10 min. And reaction was performed during 2 h at room temperature. After reaction, purification was carried out with dialysis and lyophilization.

*Characterization of β‐CD PPZ, Ad‐HAV, and Ad‐TGF*: The structures of prepared β‐CD PPZ, Ad‐HAV, and Ad‐TGF were estimated by measuring ^1^H NMR (Bruker avance III 400 MHz Fourier transform mode with DMSO‐d_6_ and CDCl_3_). The viscosity of the aqueous polymer solutions were assessed on a Brookfield RVDV‐III+ viscometer between 5 and 70 °C under a fixed shear rate of 0.1. The measurements were carried out with a set spindle speed of 0.2 rpm and with a heating rate of 0.33 °C min^−1^. Moreover, the measurement of rheology was also performed with the representative samples of T0 H0, T100 H0, and T0 H100 at the concentration of 10 wt%. The rheometer (MSC 102, Anton Paar, DE) was equipped with a politer temperature‐controlled bottom plate and a 25.0 mm parallel plate measuring system. All of the measurements were conducted with a gap length of 0.3 mm at an oscillating frequency of 1 Hz, 0.1% of the oscillating strain, and temperature in 4 and 37 °C. The storage modulus (*G*′) and loss modulus (*G*″) were calculated by the instrument's software.

*2D‐NOESY Spectra*: Spatial information was obtained from 2D‐NMR (NOESY) with a 1:1 molar mixture of β‐CD and adamantine containing peptide dissolved in D_2_O. A 2D‐NMR spectrum was recorded on a DD2 600 MHz FT NMR (Agilent Technologies).

*Dynamic Light Scattering*: The sizes of β‐CD PPZ, β‐CD PPZ included with Ad‐peptides, and Ad‐peptides alone were measured by a Zetasizer Nano ZS (Malvern Instruments Ltd., Malvern, UK) at room temperature. The final concentration of samples was 10 µg mL^−1^, and the samples were measured in triplicate.

*Transmission Electron Microscopy (TEM)*: The shapes and sizes of β‐CD PPZ, β‐CD PPZ included with Ad‐peptides, and Ad‐peptides were observed using TEM (CM30 electron microscope, Philips, CA, USA). One drop of sample solution was placed on a copper grid, and the negative staining was performed with 2 wt% uranyl acetate.

*Evaluation of β‐CD PPZ Biocompatibility*: Mesenchymal stem cells derived from Balb/c nude and mouse fibroblast cells with the consistent density (1 × 10^4^ cells per well) were seeded in 96‐well tissue culture plate (SPL, Korea). Each cell line was incubated for 24 h with the well‐dissolved β‐CD PPZ (concentration: 0–20 000 µg mL^−1^). After incubation, the spent medium was discarded and cells were washed once with DMEM and fresh PBS. After adding fresh media (200 mL per well), the 3‐(4,5‐dimethylthiazol‐2‐yl)‐2,5 diphenyltetrazolium bromide (MTT) solution (100 mg per well) was added to the cells followed by incubation for 4 h at 37 °C in a humidified atmosphere of 5% CO_2_. The formed formazan crystals were solubilized by incubating the cells with DMSO. The absorbance of the solution was measured at 570 nm, using a microplate reader (Bio‐Tek Instruments, USA). The cell viability (%) was calculated using the following formula: Cell viability (%) = [ab]test/[ab]control × 100%.

Furthermore, 3D culture of MSCs was also performed to observe the viability of MSCs in the concentration of 10 wt% hydrogel with the 100 µL volume of each well. The groups of T0 H0, T100 H0, and T0 H100 were representatively selected for this test (*n* = 3). The hydrogels encapsulating MSCs were incubated in 24‐well culture plates (SPL Life Science, KR) with cell insert (Corning, US). In the case of the CCK‐8 assay, all of the 3D hydrogels, including MSCs, were collapsed with media at 0, 7, and 14 days. Collapsed hydrogels with media were moved to 96‐well culture plates (SPL Life Sciences, KR), and 10 µL of CCK‐8 (Dojindo Molecular Technology, Inc., JP) solution was added to each well. The CCK‐8 solution contained hydrogel and was placed in the cell incubator in a humidified atmosphere at 37 °C, 5% CO_2_, for 2 h. After incubation, the absorbance was measured using a microplate reader (Bio‐Rad, Hercules, CA, US) at a wavelength of 450 nm. For live/dead assays, the culture media was removed and calcein AM/ethidium homodimer‐1 (live/dead assay kit, Thermo Fisher Scientific Inc.) dissolved in DPBS solution was substituted at 0, 7, and 14 days. All images were obtained using a confocal microscope (Zeiss LSM 800, DE) in the 3D state.

*In Vivo Artificial Tissue Generation with β‐CD PPZ/MSC/Various Ratio Combinations of Ad‐peptides*: All experiments with live mice were carried out in compliance with the relevant laws and institutional guidelines of the Institutional Animal Care and Use Committee (IACUC) in Korea Institute of Science and Technology (KIST), and IACUC approved the experiment (the approval number of 2017‐092). Balb/c nude mice (4 weeks old, 20–25 g, female) were purchased from Nara Bio Inc. (Gyeonggi‐do, Korea). Nude mice were anaesthetized with 3% isoflurane in the balanced oxygen and nitrogen. MSCs (passages below 8)/β‐CD PPZ (10 wt%)/pendently assembled 0–100% of Ad‐TGFs (T, adamantane‐PEG1000‐CESPLKRQ, 2248.70 Da) and Ad‐HAVs (H, adamantane‐PEG1000‐CLRAHAVDIN, 2399.87 Da) (**Table** **1**) were injected into subcutaneous pockets in mice on their right sides, lateral to the dorsal midline, using a syringe with a 31‐gauge needle. Each mouse received an 100 µL injection containing 2 × 10^6^ cells mixed with 10 wt% β‐CD PPZ and a regulated Ad‐RGD amount. All tissues were collected 4 weeks postinjection, and were used for histological examinations and gene analysis.

**Table 1:** Practically embraced guest molecules (Ad‐TGF and Ad‐HAV) to β‐CD PPZ hydrogel

Abbreviation	Exactly spent molecules [mg]^a)^		Abbreviation	Exactly spent molecules [mg]^a)^	
	T^b)^	H^b)^		T	H
T100 H0	1.10	0	T25 H75	0.28	0.90
T75 H25	0.83	0.30	T0 H100	0	1.20
T50 H50	0.55	0.60	T0 H0	0	0

^a)^ Exactly spent Ad‐peptides in 0.01 g of β‐CD PPZ

^b)^ T and H denote the actual spent Ad‐TGF and Ad‐HAV, respectively.

*Histological and Immunohistological Analysis*: All collected tissues were embedded in paraffin, and sectioned with a microtome (thickness 6 µm). For histological evaluations, tissue sections were deparaffinized, rehydrated, and stained with H&E, safranin‐O, von Kossa, and immunohistochemistry. For immunohistochemistry, sectioned tissues were incubated overnight 4 °C with primary antibodies: antiaggrecan (1:500, Abcam, ab3778) and anticollagen II (1:500, Abcam, ab34712). After washing three times, the slides were incubated with appropriate secondary antibodies conjugated to fluorescent dyes such as goat antimouse IgG (TRICT, Abcam, ab6786) and goat antirabbit IgG (Alexa 488, Abcam, ab150077). Images were captured using a confocal laser‐scanning microscope (Zeiss) and a bright imaging microscope (Zeiss).

*Gene Assay from Harvested In Vivo Artificial Tissue*: RNA extraction was prepared by using Trizol (Invitrogen, Carlsbad, CA). After tissues were treated with DNase (Invitrogen), 1 mg of RNA was used for cDNA synthesis (Superscript First‐strand synthesis system, GibcoBRL, Life Technologies). In brief, a reverse transcription reaction was carried out in a 20 mL mixture (1 RT buffer, 1.25 × 10^−3^ m MgCl_2_, 5 × 10^−3^ m DTT, 2.5 g random hexamer, 0.5 × 10^−3^ m each of dATP, dCTP, dGTP, and dTTP, and 50 U of Superscript II enzyme) at 42 °C. After the reverse transcription reaction, RNA was degraded by 2U of *Escherichia coli* RNase H. PCR was performed in a 50 mL reaction buffer containing 2U of Takara Taq, 1 × PCR buffer, 0.8 × 10^−3^ m dNTP mixture, and specific primers at the concentration of 100 pmol. Standard PCR conditions were as follows: 3 min at 95 °C, followed by cycles of 5 s denaturation at 95 °C, 34 s annealing at 60 °C, and 1 min extension at 72 °C. Oligonucleotides used as primers are described in **Table** **2**. The gene expression values were normalized against the housekeeping gene of β‐actin.

**Table 2:** Primer used for real time RT‐PCR

Name	Forward	Reverse
Col II	GCGGTGAGCCATGATCCGCC	GCGACTTACGGGCATCCT
Aggrecan	GAAATGACAACCCCAAGCAC	TCTCCGCTGATTTCAGTCCT
Runx2	GCGTCAACACCATCATTCTG	CAGACCAGCAGCACTCCATC
β‐actin	ACTCTTCCAGCCTTCCTTCC	ACTCGTCATACTCCTGCTTGC

## Conflict of Interest

The authors declare no conflict of interest.

## Supporting information

Supplementary
