# Supplementary material for: Fine‐Tunable and Injectable 3D Hydrogel for On‐Demand Stem Cell Niche
Source: Adv Sci (Weinh). 2019 Jul 15;6(17):1900597. doi: 10.1002/advs.201900597 (PMC6724362; doi:10.1002/advs.201900597)
Supplement: Supplementary file 1 — Supplementary [file ADVS-6-1900597-s001.pdf]

Copyright WILEY-VCH Verlag GmbH & Co. KGaA, 69469 Weinheim, Germany, 2019.

Supporting Information

for *Adv. Sci.,* DOI: 10.1002/advs.201900597

Fine-Tunable and Injectable 3D Hydrogel for On-Demand Stem Cell Niche

*Ki Hyun Hong, Young-Min Kim, and Soo-Chang Song**

Supporting Information

Fine-Tunable and Injectable 3D Hydrogel for On-Demand Stem Cell Niche

*Ki Hyun Hong, Young-Min Kim, and Soo-Chang Song**

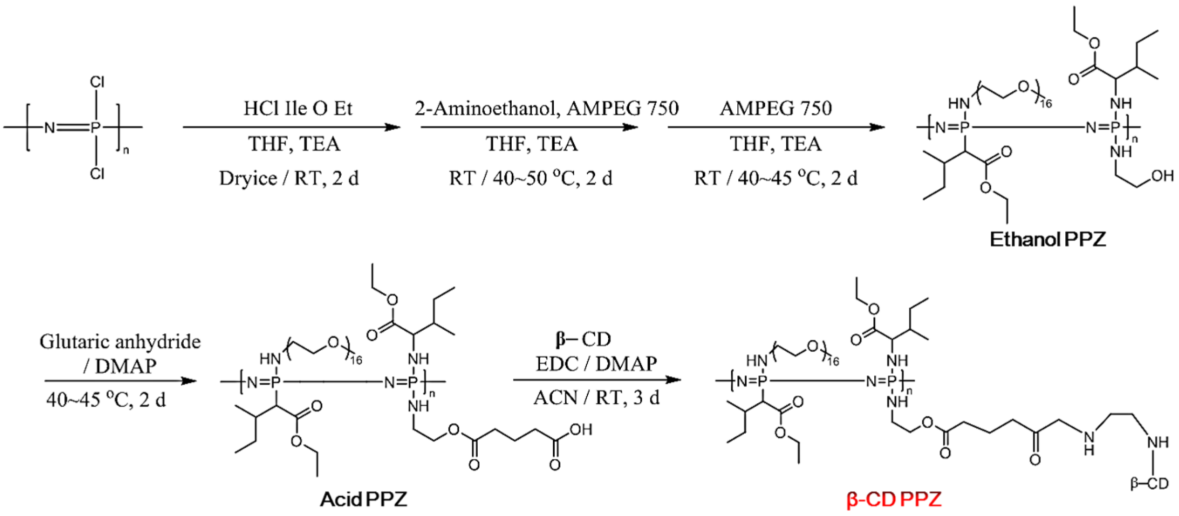

Scheme S1. Synthesis scheme for thermosensitive acid PPZ and β-CD PPZ

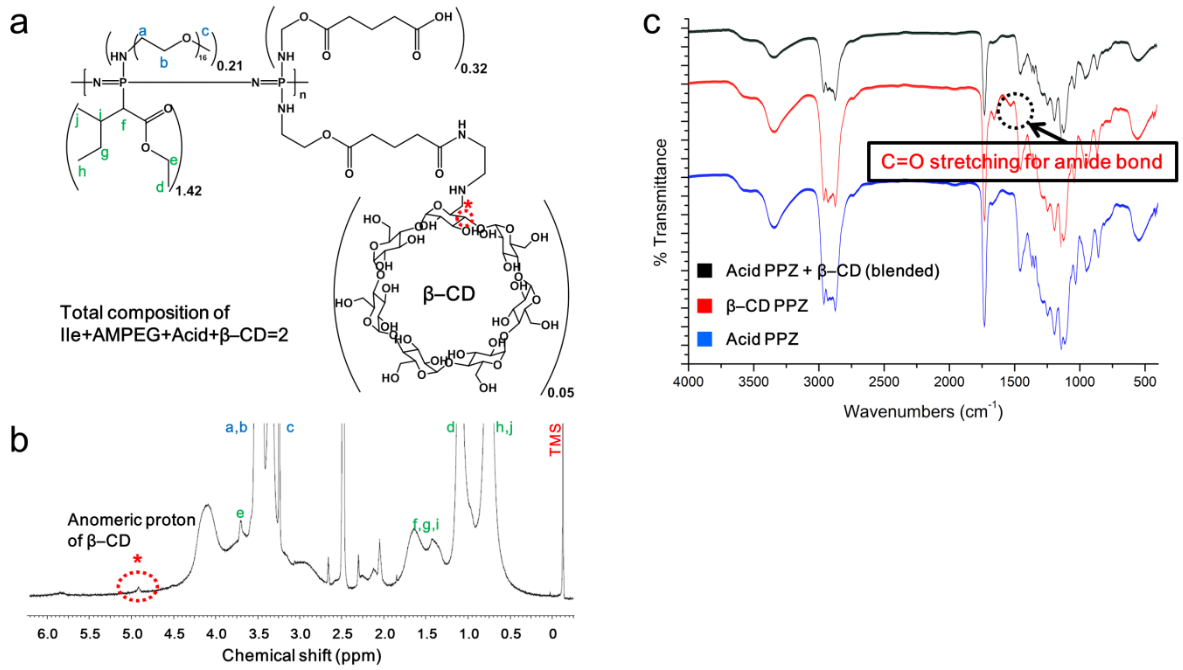

Figure S1. a) Chemical structure of β-CD PPZ. b) ^{1}H-NMR spectra result for β-CD PPZ. c) FT-IR results to prove the amide bond generation in β-CD PPZ.

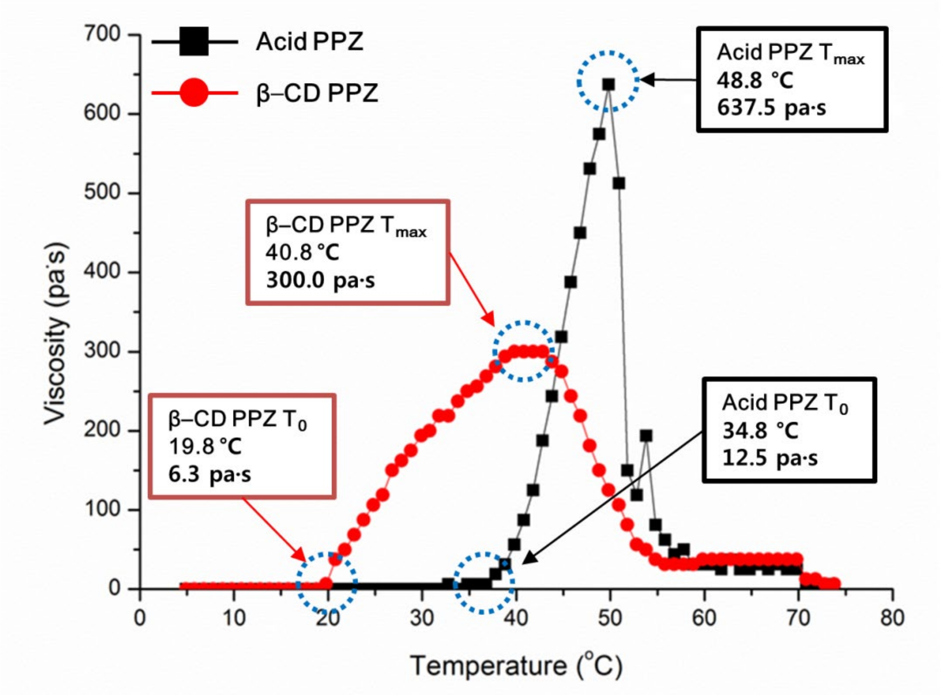

Figure S2. Thermosensitive gelation details for acid PPZ (the former β-CD PPZ before β-CD conjugation) and β-CD PPZ. T_0, a gelation starting temperature, and T_{max}, a temperature with the maximum viscosity, were indicated above, respectively.

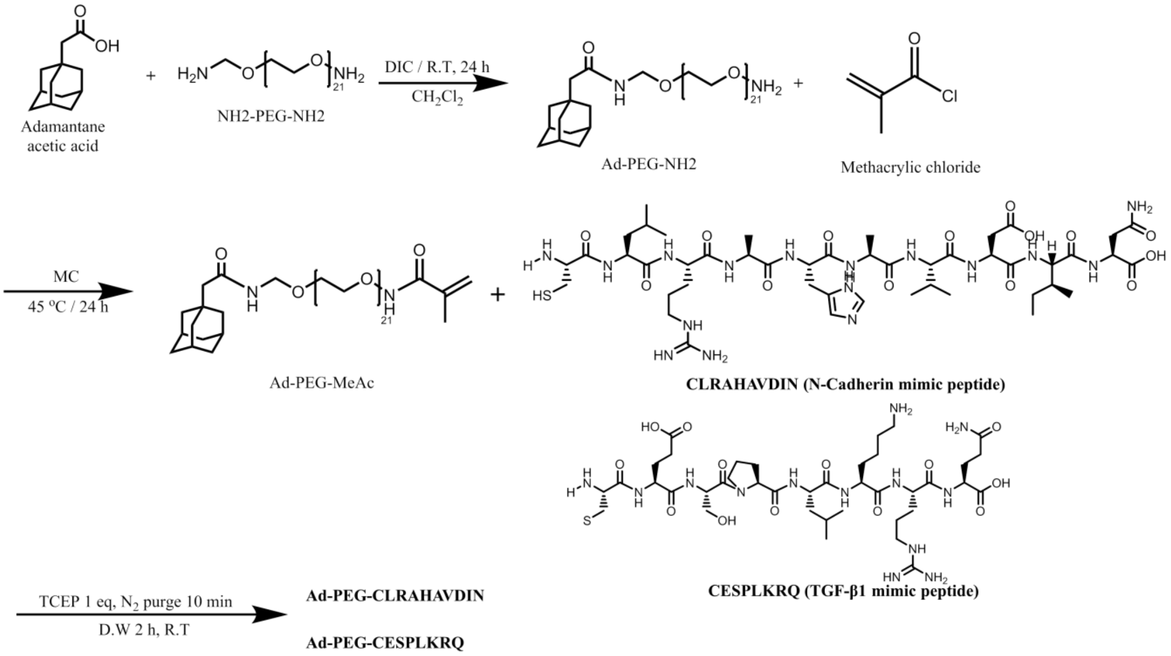

Scheme S2. Synthesis scheme for Ad-PEG-NH_2, Ad-PEG-MeAc, Ad-TGF, and Ad-HAV.

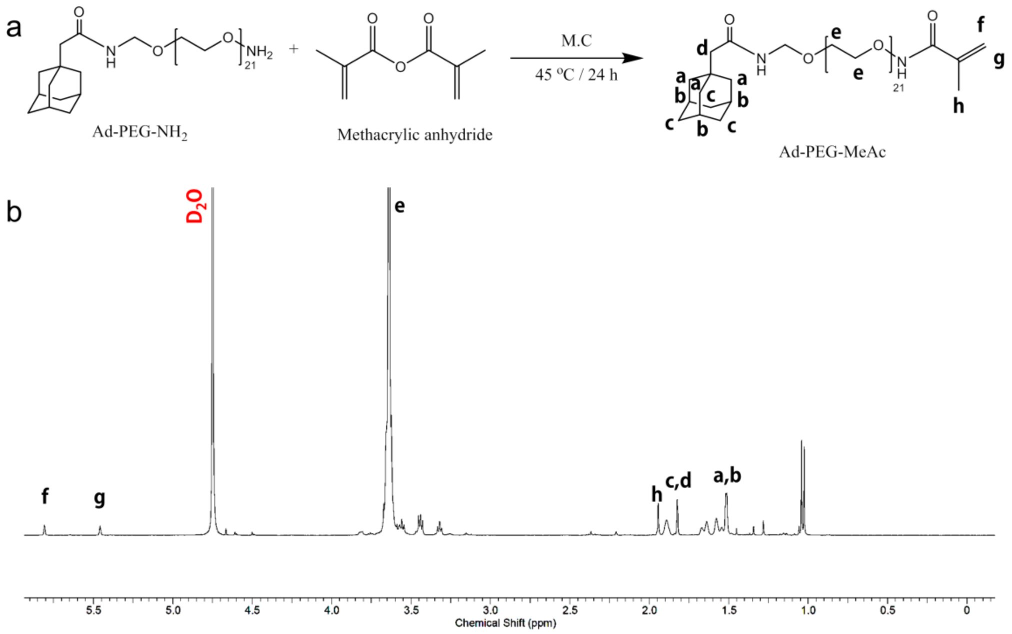

Figure S3. a) Synthesis scheme for Ad-PEG-MeAc. b) ^{1}H-NMR spectra result for Ad-MeAc. Each peaks for a-g is elucidated.

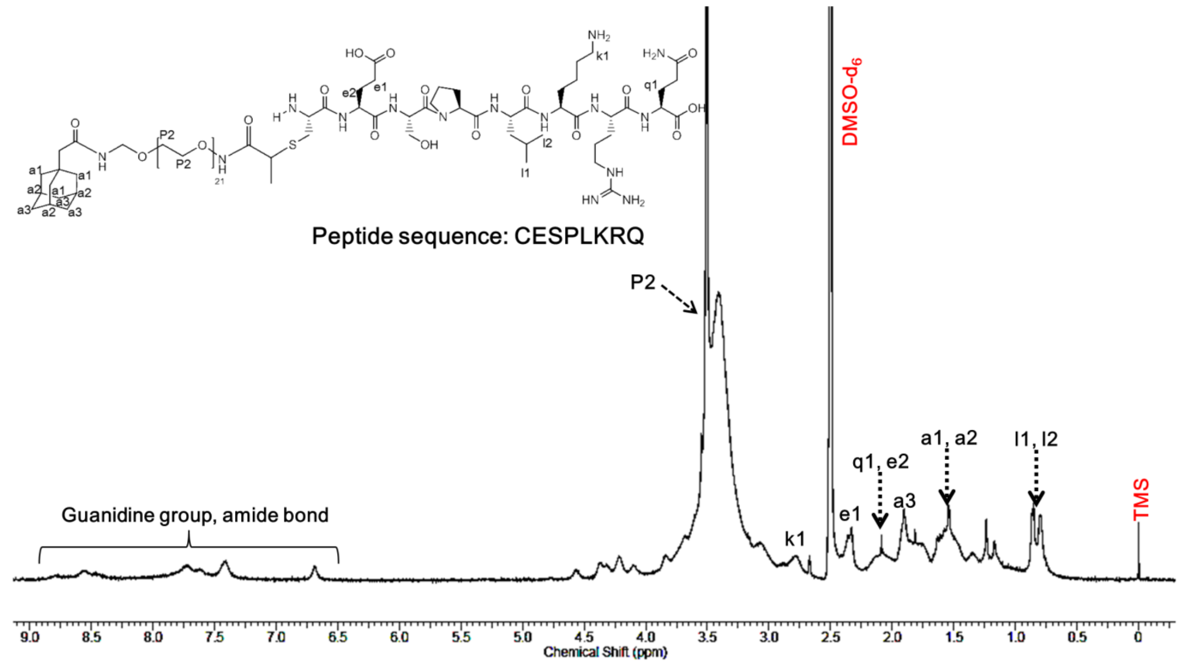

Figure S4. Chemical structure and ^{1}H-NMR result of Ad-TGF. ^{1}H-NMR spectra were measured using DMSO-d_6. TGF peptide specific peaks for guanidine, amide bond and leucine were revealed.

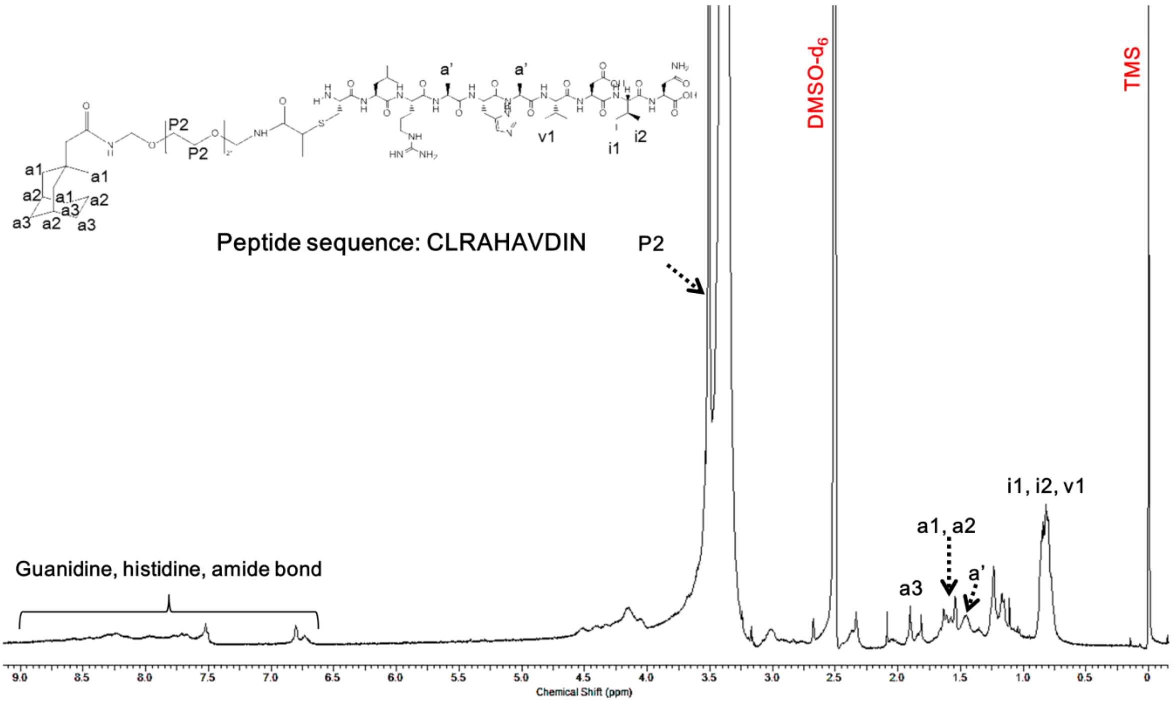

Figure S5. Chemical structure and ^{1}H-NMR result of Ad-HAV. ^{1}H-NMR spectra were measured using DMSO-d_6. HAV peptide specific peaks for guanidine, amide bond and leucine were revealed.

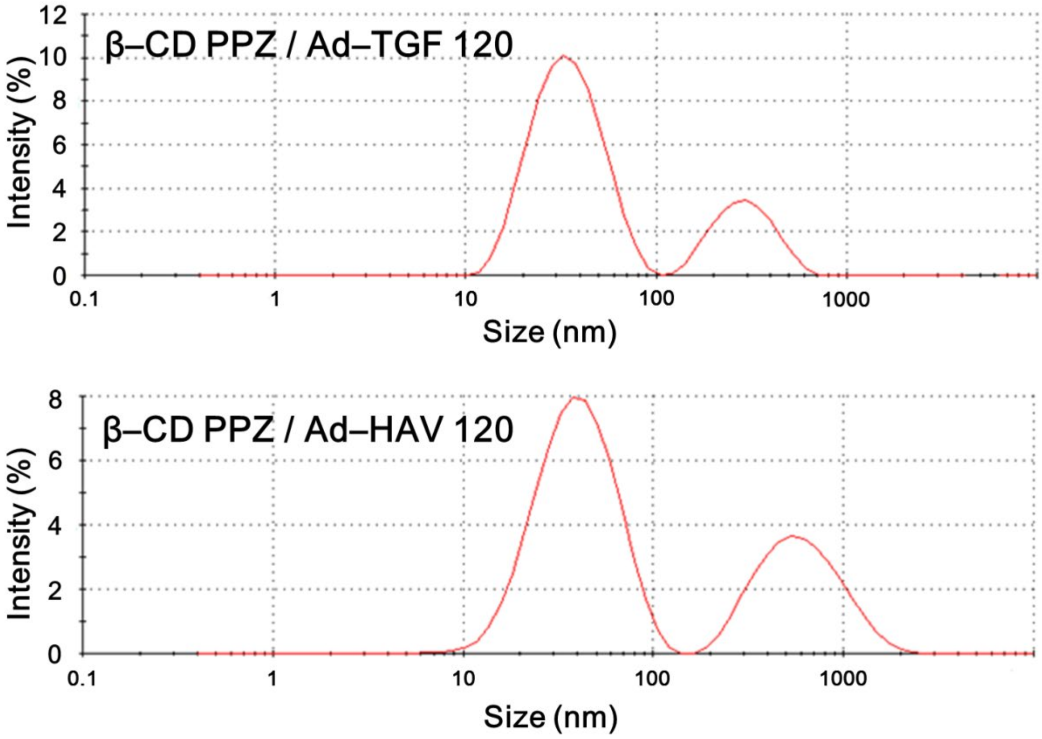

Figure S6. Size distribution results induced by excess Ad-peptides compared to β-CD PPZ. (Top: β-CD PPZ / Ad-TGF 120, bottom: β-CD PPZ / Ad-HAV 120)

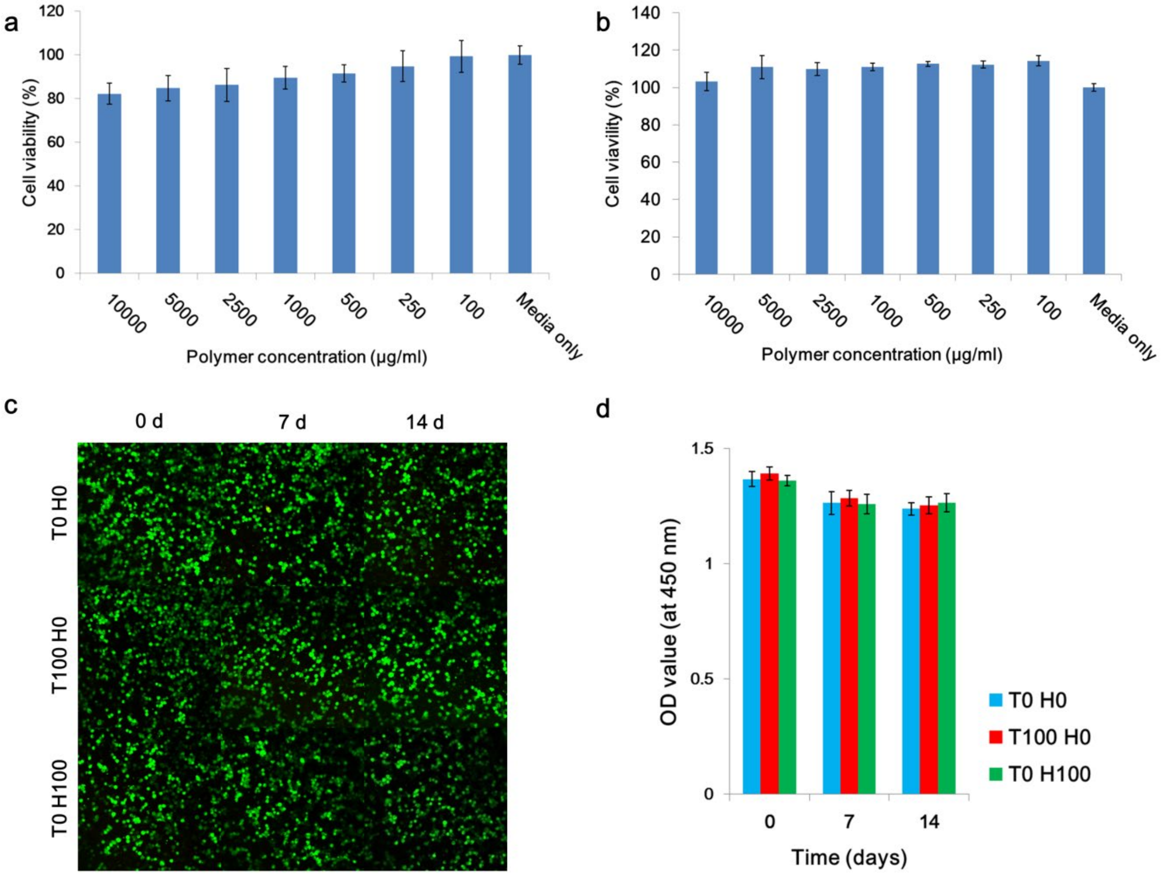

Figure S7. In vitro biocompatibility of 2D and 3D state of cells. Cytotoxicity of β-CD PPZ hydrogel under various polymer concentrations (0-10,000 μg/ml) and 2D plate attached cell lines of (a) MSCs, (b) NIH3T3 (mouse fibroblast) for 24 hours (*n* = 6). Two weeks scheduled MSCs 3D in vitro cytotoxicity with (c) live/dead assay and (d) CCK-8 assay encapsulated with the representative hydrogel groups of β-CD PPZ alone, β-CD PPZ/Ad-TGF 100, and β-CD PPZ/Ad-HAV 100 (β-CD PPZ concentration is 10 wt %, *n* = 3).
